# REMOTE COMPASSION- AND EMPATHY-BASED INTERVENTION TARGETING LONELINESS AND STRESS IN OLDER ADULTS

**DOI:** 10.1093/geroni/igad104.2731

**Published:** 2023-12-21

**Authors:** Molly Patapoff, Dylan Jester, Brent Mausbach, Barton Palmer, Danielle Glorioso

**Affiliations:** University of California San Diego, San Diego, California, United States; VA Palo Alto Health Care System, Palo Alto, California, United States; University of California, San Diego, San Diego, California, United States; University of California, San Diego, San Diego, California, United States; University of California, San Diego, San Diego, California, United States

## Abstract

Loneliness and chronic stress are prevalent issues for older adults in the United States and have been linked to decrements in mental and physical health. Prior research has suggested that increasing compassion and empathy may reduce loneliness and chronic stress in older individuals. The present study is an efficacy pilot of a remote empathy- and compassion-based intervention targeting loneliness and chronic stress in older adults, consisting of a 6-week control and 6-week intervention period. Repeated measures ANOVAs were used to compare changes in ratings and biomarkers over the 12-week period from baseline to completion of the intervention. Bonferroni-adjusted post-hoc analyses were conducted to compare changes during control and intervention periods. The full sample consisted of 43 older adults (Mean Age=76.9 years, SD=7.5), and 12 completed additional biomarker assessments. Over the 12-week period, there were significant decreases in loneliness (p=.001) and perceived stress (p=.03). Post-hoc analyses found that perceived stress significantly decreased during the intervention period (Mdifference=1.59, p=.04), but not the control period. Loneliness significantly decreased during the control period (Mdifference=2.44, p=.003) and stayed lower during the intervention period. Preliminary results revealed reduced inflammation as well as improvements in sleep quality and variability over the 12-week period. These results support the efficacy of a remote empathy- and compassion-based intervention at reducing loneliness and stress in older adults. Further investigation in a larger sample is needed to verify whether changes in biomarkers are clinically meaningful.

